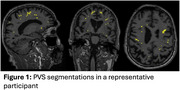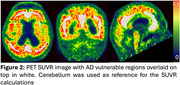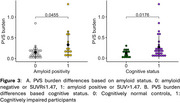# Evaluating the relationship of perivascular spaces with vascular risk factors, amyloid positivity, and cognitive impairment

**DOI:** 10.1002/alz70862_110007

**Published:** 2025-12-23

**Authors:** Swati Rane Levendovszky, Mladen Zecevic, Rebecca J Lepping, Daniel Schwartz, Lisa C Silbert, Sandra A Billinger

**Affiliations:** ^1^ University of Kansas Medical Center, Kansas City, KS USA; ^2^ University of Washington, Seattle, WA USA; ^3^ Oregon Health & Science University, Portland, OR USA

## Abstract

**Background:**

Perivascular space (PVS) burden is an emerging MRI marker of cerebrovascular disease and represents enlarged fluid‐filled spaces around blood vessels. It could also indicate stagnated CSF flow that slows amyloid clearance from the brain. Here, we sought to determine which vascular risk factor (BMI, blood pressure, pulse wave velocity) informed PVS burden. We also assessed if PVS burden was associated with amyloid positivity. Finally, we evaluated whether PVS burden was associated with cognitive status.

**Method:**

45 (74±7 years old, 24M) participants from the local ADRC, who underwent MRI and amyloid PET imaging, also participated in a study for pulse wave imaging. Pulse wave ultrasound imaging measures pressure wave propagation through large arteries and is a measure of arterial stiffness. Participants were assigned to two groups: a normal cognition group (*N* = 15) and a cognitively impaired group with MCI and AD diagnosis (*N* = 30). PVSs were detected in accordance with Schwartz et al. using T1 and FLAIR images. PVS volumes were normalized by total white matter and defined as PVS burden. Typical Florbetaben PET acquisition, processing, and analyses were performed in AD vulnerable regions with a SUVR1.47 for amyloid positivity. A lasso regression was performed to evaluate which vascular risk factors (pulse wave velocity, systolic and diastolic blood pressure, BMI – found to not be significantly correlated to each other and treated as independent in this study), predicted PVS burden. Since age and sex‐dependent differences between the amyloid‐positive (*N* = 17) and amyloid‐negative (*N* = 28) groups were not significant, PVS burden was directly compared with a t‐test. Similar comparison was performed between the two cognitive groups.

**Result:**

Figure 1 shows a representative PVS segmentation. Figure 2 shows PET SUVR map with AD vulnerable regions overlaid on top. Lasso regression found no vascular risk factors predictive of PVS burden. PVS burden was significantly higher in amyloid positive (*p* = 0.05) and cognitively impaired participants (*p* = 0.02, Figure 3).

**Conclusion:**

PVS burden was significantly greater in amyloid‐positive individuals compared to amyloid‐negative individuals, likely due to the impaired CSF flow in the PVSs associated with glymphatic clearance. Our moderate sample size could explain the absence of associations with vascular risk factors.